# Knowledge, perceptions, and health education needs regarding hepatitis B virus among HBsAg-positive pregnant women in Taiyuan, China: a cross-sectional study

**DOI:** 10.1186/s12889-025-26019-3

**Published:** 2025-12-20

**Authors:** Qiaojun Liu, Jian Shi, Ping Zhang, Gaiyan Du

**Affiliations:** 1 Health Education and Promotion Department, Taiyuan Center for Disease Control and Prevention, Taiyuan, China; 2 Taiyuan Center for Disease Control and Prevention, Taiyuan, China; 3 Public Health Department, Shanxi Bethune Hospital, Shanxi Academy of Medical Sciences, Tongji Shanxi Hospital, Third Hospital of Shanxi Medical University, Taiyuan, Shanxi 030001 China

**Keywords:** Hepatitis B virus, Mother-to-child transmission, Pregnant women, Health literacy, Misconceptions, Targeted intervention

## Abstract

**Background:**

Hepatitis B virus (HBV) infection remains a major global public health challenge, with China bearing a high burden of disease. Mother-to-child transmission (MTCT) is the primary pathway leading to chronic HBV infection. Insufficient awareness among pregnant women continues to impede effective MTCT prevention. This study aimed to assess HBV-related knowledge, perceptions, and health education needs among HBsAg-positive pregnant women in Taiyuan, China, a key population for interrupting MTCT.

**Methods:**

From March 2020 to October 2022, we conducted a hospital-based, cross-sectional survey using a structured questionnaire among 203 HBsAg-positive pregnant women recruited from five tertiary hospitals in Taiyuan. Descriptive statistics were used to summarize knowledge and perceptions. Multiple linear regression was performed to identify sociodemographic factors associated with knowledge scores.

**Results:**

Nearly half (45.3%) were first diagnosed during the current pregnancy, and the majority (84.7%) were unaware of their hepatitis B e antigen (HBeAg) status. Misconceptions were common; 41.9% incorrectly believed that HBV could be transmitted through food. Hospitals/doctors (90.1%) and the internet (85.7%) were the main information sources. Multiple linear regression identified occupation (β = − 0.215, *p* = 0.002) and urban–rural residence (β = 0.184, *p* = 0.011) as significant predictors of HBV knowledge, whereas educational level showed no significant association.

**Conclusion:**

Substantial knowledge gaps and persistent misconceptions regarding HBV transmission and prevention remain among HBsAg-positive pregnant women in Taiyuan. The stronger influence of occupation and place of residence, compared with formal education, underscores the need for tailored and context-specific health education programs. Therefore, interventions should be tailored to occupational settings and rural–urban contexts, and antenatal counseling must be strengthened to address specific misconceptions. These targeted efforts are essential to improve maternal awareness and support China’s efforts to eliminate MTCT of HBV.

**Supplementary Information:**

The online version contains supplementary material available at 10.1186/s12889-025-26019-3.

## Introduction

Hepatitis B virus (HBV) infection continues to be a significant global public health challenge. The World Health Organization (WHO) estimates that 296 million people worldwide live with chronic HBV infection, and approximately 820,000 deaths occur annually resulting from HBV – related complications such as cirrhosis, liver failure, and hepatocellular carcinoma. Despite the availability of an effective vaccine, HBV remains highly endemic in certain regions, notably in East Asia and sub-Saharan Africa, where the majority of infections occur through MTCT or perinatal exposure [1, 2].

In regions with high HBV prevalence, MTCT accounts for a substantial proportion of new chronic infections. Without prophylactic intervention, up to 90% of infants born to mothers who are positive for both hepatitis B surface antigen (HBsAg) and hepatitis B e antigen (HBeAg) will develop chronic HBV infection [3–5]. Consequently, vertical transmission contributes disproportionately to the long-term reservoir of people with chronic HBV infection, since infections acquired at birth progress to chronicity at a much higher rate than those acquired later in life [6].

China has long been recognized as a country with a high burden of HBV infection. However, prevalence has declined significantly over recent decades, largely due to nationwide vaccination efforts [7]. Following the introduction of the hepatitis B vaccination program in 1992, and especially after the implementation of the universal birth-dose policy in 2002, the prevalence of HBsAg in the general population decreased from 9.8% in 1992 to 6.1% in 2006 and to approximately 5% in 2014. Among children under five years of age, prevalence decreased from 9.7% to less than 1% by 2020 [8, 9].

Historically, Shanxi Province in north-central China reported an HBsAg positivity rate of 10 to 12% among pregnant women in the early 2000s, a level higher than the national average [8]. Since 2015, the nationwide expansion of maternal HBV screening and standardized perinatal prevention protocols has led to notable improvements. Between 2015 and 2020, national HBsAg positivity among pregnant women declined from 7.9% to 6.3%, and the rate in Shanxi fell even more sharply to 1.9% in 2020. This reduction places Shanxi among the regions with the lowest prevalence in the country [10]. Taiyuan, the provincial capital, has shown the same downward trend. Recent surveillance data indicate that HBsAg prevalence among pregnant women in Taiyuan is now below 2%, which classifies the area as one of low endemicity [11]. These achievements are largely attributable to near-universal antenatal screening coverage, timely administration of the hepatitis B birth dose, and the provision of hepatitis B immunoglobulin (HBIG) for infants born to HBsAg-positive mothers.

Despite these advances, the risk of MTCT has not been completely eliminated. Breakthrough infections still occur among infants, even when timely vaccination and HBIG are administered, particularly in cases where maternal HBV DNA levels are high [12–14]. For this reason, maternal awareness, timely diagnosis, and adherence to recommended prevention measures remain essential for further reducing MTCT risk.

The implications of HBV infection during pregnancy extend beyond vertical transmission. It also affects maternal health and pregnancy outcomes. Prior research has examined the association between maternal HBsAg positivity and adverse pregnancy outcomes, although results remain inconsistent [15–17]. Some studies have suggested an increased risk of complications such as gestational diabetes mellitus (GDM). For example, Liu et al. reported a higher risk of preterm delivery among HBsAg-positive mothers after adjustment for confounders [18], and Sirilert et al. observed similar findings among HBeAg-positive individuals [19]. Additionally, HBV reactivation may occur during pregnancy or postpartum due to immune system fluctuations or the use of immunosuppressive therapy [20]. These findings highlight the importance of enhanced monitoring, patient counseling, and targeted interventions throughout pregnancy and after delivery.

Eliminating MTCT is essential from a public health perspective and is a core target of the WHO’s goal to eliminate viral hepatitis as a public health threat by 2030 [21]. China has set parallel national goals emphasizing universal maternal screening, antiviral treatment when indicated, and strengthened health education within routine perinatal care. Considerable progress has been achieved. A large nationwide study involving more than nine million pregnant women demonstrated that integrated interventions, including universal screening, timely birth-dose vaccination, and antiviral therapy for mothers with high viral loads, reduced the MTCT rate to 1.08% [22]. Continued progress, however, requires not only medical interventions but also adequate awareness and adherence among pregnant women.

Although China has made substantial gains in reducing HBV prevalence, significant gaps remain in maternal knowledge and health-seeking behaviors, especially among HBsAg-positive pregnant women. Most existing studies have focused on the general pregnant population, while fewer have examined the subgroup of HBsAg-positive women, who play a central role in preventing MTCT.

A major policy change in 2015 substantially reshaped HBV screening among pregnant women in China. The National Health Commission integrated HBV screening into the national program for the prevention of MTCT of HIV, syphilis, and HBV. The policy mandated universal and free HBV screening for all pregnant women, including internal migrants, across all counties in China [23, 24]. This strategy has significantly increased the identification of previously undiagnosed HBV infections. As a result, a growing number of women receive their first HBV diagnosis during pregnancy. While this reflects a major public health success, it also introduces new challenges related to understanding the characteristics, perceptions, and educational needs of these women, and to ensuring timely linkage to care and antiviral treatment.

The integration of universal and free screening into routine antenatal care has created a unique population of newly diagnosed HBsAg-positive pregnant women. These women must cope with a new chronic disease diagnosis while managing the demands of pregnancy, which creates distinct challenges for risk communication and health education. Although screening coverage is high, the specific knowledge levels, misconceptions, and information needs of these women remain poorly understood. In line with this, prior studies have shown that inadequate knowledge, persistent misconceptions, and disparities between urban and rural populations continue to hinder effective MTCT prevention. These gaps impede timely screening, reduce acceptance of antiviral treatment, and undermine adherence to neonatal immunization schedules.

The present study was designed to address this evidence gap. We investigated HBV-related knowledge, perceptions, information sources, and educational needs among HBsAg-positive pregnant women in Taiyuan, Shanxi Province, in the period following the 2015 policy implementation. Unlike most previous research, which has focused on the general pregnant population, this study specifically targets HBsAg-positive women, who are central to MTCT prevention. The objective was to identify persistent knowledge gaps and the sociodemographic factors associated with them, with particular attention to the needs of women who were newly diagnosed through the screening program. The findings aim to support the design of targeted educational strategies that strengthen the continuum of perinatal HBV prevention and accelerate progress toward China’s national goal of eliminating MTCT of HBV.

## Materials and methods

### Study design and setting

A cross-sectional study was conducted in Taiyuan, Shanxi Province, China, from March 2020 to October 2022. Five tertiary obstetric hospitals participated in the study. The objective was to assess HBV-related knowledge and health education needs among pregnant women who tested positive for HBsAg.

### Study population

The study population consisted of pregnant women attending routine antenatal visits in participating hospitals. Women were eligible if they met all of the following criteria: (1) documented HBsAg positivity in their medical records; (2) singleton pregnancy; (3) gestational age of at least 12 weeks; (4) ability to understand and complete the questionnaire independently; and (5) provision of written informed consent.

Exclusion criteria were: (1) coinfection with HIV, HCV, or other severe comorbidities such as decompensated cirrhosis; (2) cognitive impairment or psychiatric disorders that could interfere with participation; (3) refusal to participate.

For analysis, participants were categorized into two groups based on the timing of their first HBV diagnosis: the newly diagnosed group, defined as women first diagnosed during the current pregnancy, and the previously aware group, defined as women who had known their HBV status prior to the current pregnancy. This grouping was used for the comparisons shown in Table 6.

### Sample size and sampling procedure

A total of 412 HBsAg-positive pregnant women were identified through routine prenatal screening in the five participating hospitals during the study period. To enhance representativeness and reduce selection bias, a list-based simple random sampling method was used.

The procedure was as follows:

A researcher who had no direct contact with participants obtained the complete list of all 412 eligible women.

A random sequence was generated using the sample() function in R software (version 3.6.1).

Eligible women were invited sequentially according to this random order during antenatal visits.

Recruitment continued until the target sample size of 203 was reached.

Of the 412 eligible women, 203 completed the questionnaire, yielding a response rate of 49.3%. This sampling approach ensured that all eligible women had an equal probability of being selected.

**Fig. 1:**
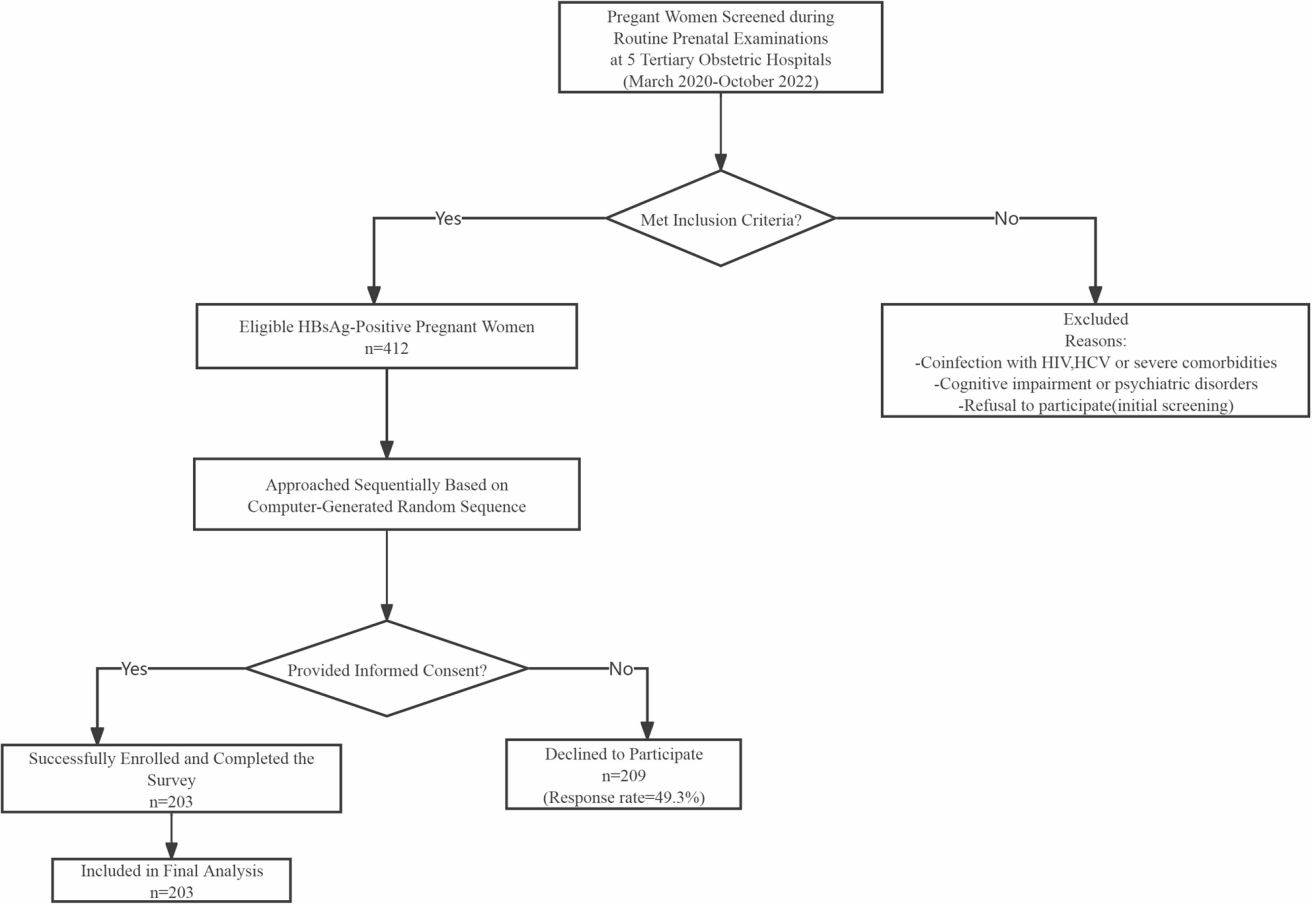
The flow diagram of participants enrollment

Figure 1 presents the participant recruitment flow.

### Data collection tool

Data were collected using a structured, self-administered questionnaire. The present analysis uses data from selected sections of a larger parent study. Only the sections on sociodemographic characteristics, HBV-related knowledge, and health education needs were included. Components related to laboratory data, such as HBeAg status or HBV DNA levels, were not used in this analysis. The questionnaire consisted of three components: sociodemographic characteristics; HBV-related knowledge, comprising 16 items on transmission routes, disease severity, and prevention; and health education needs, comprising 5 items on preferred sources and modes of information.

A total knowledge score was calculated by summing the correct responses to the 16 items. Each correct response received 1 point, and incorrect or unknown responses received 0 points, giving a possible score range from 0 to 16. The internal consistency of the scale was satisfactory with a Cronbach’s alpha of 0.78 [25], indicating acceptable reliability for the knowledge assessment. Content validity was established through a literature review and expert consultation following established methods for instrument development [26].

### Sample size

The sample size was calculated using the standard formula for cross-sectional studies that estimate a proportion: $\mathrm{n}=\frac{Z^{2}_{\upalpha/2}\times\mathrm{p}\times\left(1-\mathrm{p}\right)}{\mathrm{d}^2}$

The parameters were set as follows: Z_α/2_ = 1.96 for a 95% confidence level, *p* = 0.5 as a conservative estimate of the proportion of pregnant women with insufficient HBV knowledge. This value was selected for two reasons: (1) it is conservative and provides the maximum sample size estimate when the true proportion is uncertain; and (2) it is supported by a previous study in a similar population of HBsAg-positive pregnant women in China, which reported a high prevalence of knowledge gaps [27], and d = 0.07 as the acceptable margin of error.

This calculation yielded a minimum required sample size of 196. The final target was increased to 203 to account for possible non-response and to ensure adequate sample size for multiple regression, based on the general rule of 10 to 15 participants per predictor variable [28].

### Data collection procedure

During routine antenatal visits, eligible women were identified by trained research staff. After the study purpose was explained and written informed consent was obtained, each participant received a printed card that contained a QR code and a URL linking to the questionnaire hosted on the Wenjuanxing platform. Participants completed the questionnaire on their own smartphones while in the clinic. Staff were available to assist with technical issues if needed. Completed responses were uploaded directly to the secure Wenjuanxing server.

The platform included quality control features such as mandatory responses, logical skip patterns, and IP address restrictions. This digital data collection process reduced transcription errors and suited the high digital literacy level of the participants.

### Data quality assurance

The research team conducted daily checks to ensure the completeness and accuracy of collected data. The questionnaire had been pretested in a comparable population, and adjustments were made based on the feedback received.

### Data analysis

Statistical analyses were performed using R software (version 3.6.1). Descriptive statistics were used to summarize characteristics, knowledge levels, perceptions, and information sources. Continuous variables were reported as means and standard deviations, and categorical variables were reported as frequencies and percentages with 95% confidence intervals.

Differences in categorical variables were assessed using Pearson’s chi-square tests or chi-square goodness-of-fit tests when appropriate. Binomial tests were used to determine whether the proportion of correct answers for each knowledge item differed significantly from 50%.

### Factors associated with HBV knowledge

To assess factors associated with HBV knowledge, two primary analyses were conducted. First, multiple linear regression was used with the total knowledge score as a continuous dependent variable. Second, logistic regression analysis was employed using the median knowledge score as the cutoff to classify participants into high- and low-knowledge groups. For both regression analyses, odds ratios (ORs) and 95% confidence intervals (CIs) were estimated to determine the independent effects of sociodemographic characteristics.

A two-tailed p-value of less than 0.05 was considered statistically significant.

### Ethical considerations

The study was approved by the Scientific Research Ethics Review Committee of the Taiyuan Center for Disease Control and Prevention (Approval No. 2020001; approved on October 15, 2020). Prior to participation, written informed consent was obtained from all individual participants. The study adhered to the principles of the Declaration of Helsinki and followed national guidelines for biomedical research involving human participants.

## Results

### Participant characteristics

A total of 203 HBsAg-positive pregnant women were recruited from a sampling frame of 412 eligible participants, yielding a response rate of 49.3%. The mean age was 28.84 ± 5.01 years, and most participants (91.6%) were between 20 and 35 years of age. Educational attainment varied: 0.4% had completed primary school, 3.9% junior high school, 21.1% high school, 28.0% college, 43.3% university, and 2.9% held a master’s degree or higher. Most participants resided in urban areas (83.7%) and were primiparous (60%).

Occupational categories included administrative staff such as government or civil service employees, workers, teachers, farmers, and medical staff (29.6%); commercial service workers, houseworkers, and unemployed individuals accounted for 31.5%; The remaining 38.9% were categorized as “other”, including emerging, hybrid, or non-traditional occupations such as freelancers, IT or technology workers, digital content creators, cross-sector service providers, and individuals with multiple or fluid occupational identities.

A substantial proportion of participants (45.3%, 92/203) were first diagnosed with HBV during the current prenatal examination. Among primiparous women, 9.1% (19/203) had known their HBV-positive status for more than five years. Most participants (84.7%, 172/203) were unsure of their HBeAg status, although 85.2% (173/203) reported knowing their liver function status. Prior to the current pregnancy, 70.9% (144/203) had never undergone HBV-related testing. Regarding family history, 55.7% (113/203) were unsure whether their parents or siblings had HBV infection, while 44.3% (90/203) confirmed a family history. In terms of vaccination, 79.8% (162/203) reported that their family members had received HBV vaccination.

### Factors associated with first HBV diagnosis during the current pregnancy

For this analysis, the dependent variable was the interval since first HBV diagnosis, measured in years. It was calculated by subtracting the year of the first HBsAg-positive diagnosis from the survey year. A shorter interval indicated a more recent diagnosis, such as diagnosis during the current pregnancy, while a longer interval reflected an earlier diagnosis. This analysis was conducted to identify factors associated with the timing of diagnosis. The overall model was statistically significant (F(8, 194) = 3.095, *p* < 0.05).

As shown in Table 1, having health insurance (β = 0.236, *p* = 0.001) was significantly associated with the timing of diagnosis, specifically a diagnosis occurring during the current pregnancy. Residence in an urban area compared with rural areas or villages was also significantly associated with an earlier diagnosis (β = 0.152, *p* = 0.049). In contrast, age, education level, occupation, parity, and monthly income showed no significant association with diagnosis timing.

**Table 1:** Factors associated with the interval since first HBV diagnosis (Linear Regression Analysis)

Variable	Category	β	95% CI	t-value	*p*-value
Age		-0.022	(0.000, 0.000)	-0.191	0.849
Education	High school or below (Ref)				
	Technical school/College	0.061	(-0.001, 0.002)	0.513	0.608
	Bachelor’s or above	0.039	(-0.001, 0.002)	0.389	0.698
Residence	Rural/Village (Ref)				
	Urban	0.152	(0.000, 0.003)	1.981	**0.049**
Health Insurance	No(Ref)				
	Yes	0.236	(0.001, 0.004)	3.222	**< 0.01**
Occupation	Others(Ref)				
	Commercial/Housewife/Unemployed	0.132	(0.000, 0.002)	1.673	0.096
	Cadre/Worker/Teacher/Farmer/Medical	0.042	(-0.001, 0.001)	0.518	0.605
Parity	Primipara (Ref)				
	Multipara	0.000	(-0.001, 0.001)	0.003	0.997

β: Standardized regression coefficient

Definition of Dependent Variable (Interval Since First Diagnosis): This dependent variable represents the time elapsed (in years) since the participant’s first HBsAg-positive diagnosis, calculated as (Survey Year) - (Year of First Diagnosis). A smaller value indicates a more recent diagnosis. For instance, a value of 0.5 would indicate a diagnosis occurred approximately six months prior to the survey, while a value of 0 would indicate diagnosis during the current pregnancy. A positive regression coefficient indicates an association with a longer interval since diagnosis

Reference Group: The reference group for each categorical variable is indicated as “Ref”

### Sources of HBV knowledge

Hospitals/doctors, internet searches, and social media were the predominant sources of HBV information, each with utilization rates exceeding 80% (Table 2). In contrast, structured channels such as community lectures and government campaigns were underutilized, with usage below 16%. A Pearson chi-square goodness-of-fit test indicated that the distribution of responses differed significantly from a uniform distribution (χ² = 477.0, df = 6, *p* < 0.001), demonstrating substantial variation in the popularity of information sources.

**Table 2:** Sources of HBV-related knowledge (Multiple responses, *n* = 203)

Source	*n* (%)	95% CI*
Hospital/Doctors	183 (90.1)	85.3–93.5
Internet Search	174 (85.7)	80.2–89.9
Social Media	166 (81.8)	75.9–86.5
Community Lectures	32 (15.8)	11.4–21.4
Friends/Colleagues	24 (11.8)	8.1–17.0
Books/Magazines	17 (8.4)	5.3–13.0
NGOs	8 (3.9)	2.0–7.6

* *CI* confidence interval

### Perceptions of HBV infectivity and severity

Perceptions of infectivity varied significantly (χ² = 128.4, df = 4, *p* < 0.001). A total of 73.4% considered HBV to be very or quite infectious, while 7.9% viewed it as less infectious. Similarly, 64.5% perceived the disease as very or quite serious, whereas 35.5% regarded it as moderately serious or not serious at all (χ² = 56.6, df = 3, *p* < 0.001). Detailed results are presented in Table 3.

**Table 3:** Perceptions of HBV infectivity and severity (*n* = 203)

Category	*n* (%)	95% CI
Infectivity		
Very infectious	93 (45.8)	39.1–52.7
Quite infectious	56 (27.6)	21.9–34.1
Moderately infectious	38 (18.7)	14.0–24.6
Not very infectious	15 (7.4)	4.5–11.8
Not at all infectious	1 (0.5)	0.1–2.7
Severity		
Very serious	90 (44.3)	37.7–51.2
Quite serious	41 (20.2)	15.3–26.2
Moderately serious	56 (27.6)	21.9–34.1
Not serious	16 (7.9)	4.9–12.4

### Knowledge of HBV transmission routes

Most respondents correctly identified the primary HBV transmission routes, including blood exposure (88.2%), MTCT (60.1%), and sexual contact (53.7%). However, misconceptions remained, with 41.9% incorrectly believing that HBV could be transmitted via food and 9.4% believing transmission could occur through air (Table 4). Binomial tests indicated that recognition rates for blood exposure, mother-to-child transmission, and the incorrect beliefs about food and air transmission were statistically significant (*p* < 0.05).

**Table 4:** Knowledge of HBV transmission routes (multiple responses, *n* = 203)

Transmission Route	Correct (%)	Incorrect (%)	*p*-value*
Blood exposure	179 (88.2)	24 (11.8)	< 0.001
Mother-to-child	122 (60.1)	81 (39.9)	0.005
Sexual contact	109 (53.7)	94 (46.3)	0.326
Food	118 (58.1)	85 (41.9)	< 0.001
Air	184 (90.6)	19 (9.4)	< 0.001

*p-values from binomial tests against null hypothesis of a 50% correct response rate

### Knowledge of HBV prevention methods

Vaccination was the most widely recognized preventive measure (100%), followed by avoidance of high-risk behaviors (90.1%) and personal hygiene (89.2%) (Table 5). A Pearson chi-square test showed a significant difference in recognition rates across the three preventive methods (χ² = 22.709, df = 2, *p* < 0.001).

**Table 5:** Knowledge of HBV prevention methods (multiple responses, *n* = 203)

Prevention Method	*n* (%)	95% CI
Vaccination*	203(100.0)	98.1–100.0
Avoiding high-risk behavior	183(90.1)	85.3–93.5
Personal hygiene	181(89.2)	84.1–92.7

*Statistical testing was not performed for vaccination because recognition was complete

### Demographic correlates of knowledge

Multiple linear regression analysis was performed using the total knowledge score as the dependent variable. Significant associations were identified with occupation (β = −0.215, *p* = 0.002) and residence (urban versus rural) (β = 0.184, *p* = 0.011). No significant associations were found for education level, age, parity, or health insurance status (all *p* > 0.05).

To further examine knowledge correlates, participants were stratified by the timing of diagnosis. As shown in Table 6, newly diagnosed women aged 30 years or older had significantly higher knowledge scores than their younger counterparts (*p* = 0.047). A similar age-related pattern was observed among previously aware women (*p* = 0.043). In contrast, the association between education level and knowledge score was inconsistent and not statistically significant within either diagnostic group (*p* > 0.05 for both).

**Table 6:** Comparison of HBV knowledge scores by timing of diagnosis and sociodemographic characteristics

Variable	Category	Newly Diagnosed (*n* = 92)*	Previously Aware (*n* = 111)*	*p* (Between Groups)^b^
Age Group				0.004
	< 30 years	7 (7, 8)	7 (7, 8)	
	≥ 30 years	8 (7, 8)	8 (7, 8)	
p (Within Group)^c^		0.047	0.043	
Education Level				0.159
	High school or below	7 (7, 8)	8 (7, 8)	
	College	7 (7, 8)	7 (7, 8)	
	Bachelor or above	8 (7, 9)	8 (7, 8)	
p (Within Group)^c^		0.075	0.275	
Residence				0.921
	Rural/Urban village	8 (7, 9)	7 (7, 8)	
	Urban	8 (7, 8)	8 (7, 8)	
p (Within Group)^c^		0.261	0.384	
Health Insurance				0.392
	No	8 (7, 9)	7 (6.25, 8)	
	Yes	8 (7, 8)	8 (7, 8)	
p (Within Group)^c^		0.217	0.18	
Occupation				0.138
	Others	7.5 (7, 8)	7 (7, 8)	
	Commercial/Housewife/Unemployed	8 (7, 9)	7 (7, 8)	
	Cadre/Worker/Teacher/Farmer/Medical	8 (7, 8)	8 (7, 8)	
p (Within Group)^c^		0.289	0.521	
Age (Continuous)	r (*p*-value)^d^	0.222 (0.033)	0.129 (0.178)	

^a^ Between-group p-value: Mann-Whitney U test comparing Newly Diagnosed vs. PreviouslyAware groups within each sociodemographic category

^b^ Within-group p-value: Kruskal-Wallis test (for ≥ 3 categories) or Mann-Whitney U test (for 2categories) comparing knowledge scores across categories within each diagnostic group

^c^ Spearman’s rank correlation coefficient (r) and its p-value for the association betweencontinuous age and knowledge score

^*^ For comparisons between two categories (e.g., Age Group, Health Insurance), the Mann-Whitney U test was used

^*^ For comparisons across three or more categories (e.g., Education Level, Occupation), the Kruskal-Wallis test was used

### Summary of key findings

In summary, our results highlight five key aspects:

1. 45.3% of women were first diagnosed during pregnancy, indicating delayed screening.
2. 84.7% were unaware of their HBeAg status, a crucial risk marker, although 85.2% were aware of their liver function status.
3. Participants heavily relied on hospitals/doctors (90.1%), the internet (85.7%), and social media (81.8%), whereas only 15.8% used structured sources like community lectures.
4. Misconceptions about transmission persisted, with 41.9% wrongly believing in foodborne transmission.
5. Occupation and urban-rural residence significantly predicted knowledge, but education level did not significantly predict knowledge.

## Discussion

This study provides a detailed assessment of HBV-related knowledge and perceptions among a key population for MTCT prevention, namely HBsAg-positive pregnant women in Taiyuan, China. Our findings reveal multiple challenges, including knowledge gaps, misconceptions, and systemic barriers that may pose a challenge to progress toward eliminating MTCT within the HBV prevention continuum. The success of universal screening is reflected in the high proportion (45.3%) of women first diagnosed during pregnancy, highlighting earlier gaps in case identification. Among those newly identified, limited awareness of HBeAg status was associated with reduced access to risk-stratified management. Without adequate guidance, participants frequently relied on digital sources, which can improve access but may also expose them to misinformation and stigmatizing narratives. These results indicate that gaps remain in the current HBV MTCT elimination strategy and that variations in knowledge are affected more by environmental and occupational factors than by formal education alone. Integrated clinical and educational interventions are therefore needed to address these deficiencies. In the sections that follow, we examine these major findings, discuss their implications, and consider their relevance for public health practice.

### Universal prenatal screening as an effective safety net and associated socioeconomic factors

The finding that 45.3% of participants were first diagnosed during pregnancy reflects the influence of the 2015 national policy mandating universal and free antenatal HBV screening. This program serves as a robust safety net by systematically identifying women with chronic HBV infection who may otherwise remain undiagnosed due to a lack of symptoms. The high proportion of new diagnoses during pregnancy is therefore an expected consequence of a public health strategy that prioritizes comprehensive case detection within antenatal care.

Further analysis revealed significant socioeconomic differences related to the timing of diagnosis. As shown in Table 2, lack of health insurance strongly predicted first-time diagnosis during pregnancy. This pattern suggests that while the national policy eliminates direct financial barriers for screening, broader economic and access-related challenges still influence when women enter the HBV care cascade. Consequently, prenatal screening is particularly crucial for socially and economically disadvantaged groups with limited opportunities for voluntary health assessments before pregnancy. In this context, universal screening ensures timely identification at a point when interventions can most effectively prevent MTCT. This high detection rate underscores the success of the 2015 universal screening policy in identifying previously undiagnosed infections and providing a critical opportunity for intervention.

### Inadequate serological profiling: a barrier to risk-stratified care

The overwhelmingly high level of uncertainty regarding HBeAg status (84.7%) is concerning, particularly given that most participants reported awareness of their liver function results. HBeAg is a central predictor of MTCT risk [29, 30], and the discrepancy likely reflects difficulties in patient-provider communication and the complexity of HBV serology for laypersons. Understanding HBV serological markers requires familiarity with multiple indicators and their combinations, making concepts such as “Large Three Positive” and “Small Three Positive” challenging to recall. In contrast, liver function results are typically communicated in a simpler binary format.

This suggests that standard antenatal care may lack effective counseling. Providing laboratory results alone is insufficient unless patients understand their meaning. The poor understanding of HBeAg status indicates that its clinical significance is not being effectively communicated or retained, illustrating a gap between diagnosis and informed, risk-stratified care.

Addressing this issue requires interventions directed at both patients and healthcare providers. Counseling should clearly explain the implications of the results using accessible language, such as how specific markers relate to the infant’s risk and the need for antiviral therapy. Visual aids and simplified explanations may improve comprehension. Greater emphasis on the most clinically relevant markers, such as HBeAg, may also enhance retention.

This gap points to a need for counseling that extends beyond vaccination to include viral load monitoring and the indications, timing, and benefits of antiviral therapy.

It is important to recognize a limitation of our knowledge assessment. Although the questionnaire evaluated general HBV knowledge on transmission and prevention, it did not assess understanding of the broader MTCT prophylaxis protocol, particularly the role of antiviral therapy during pregnancy. Given the central importance of antiviral treatment for women with high viral loads, the omission likely means that participants’ overall preparedness for MTCT prevention is overestimated. The high rate of unawareness regarding HBeAg status further underscores this issue. Future research should therefore incorporate questions on antiviral therapy to more comprehensively assess readiness for participation in MTCT prevention.

### Information sources and the digital divide

Hospital/doctors (90.1%), internet searches (85.7%), and social media (81.8%) were the most common information sources, whereas NGO campaigns were used infrequently (3.9%). Heavy reliance on digital and clinical sources has both advantages and drawbacks. Although these platforms are accessible, they also contain substantial misinformation [31], which may contribute to misconceptions identified in our study, such as the belief that HBV is transmitted through food (41.9%). This highlights the importance of digital health literacy, meaning the ability to locate, understand, and appraise online health information. The underuse of structured community channels may reflect limited availability and the influence of HBV-related stigma. Misconceptions about transmission can reinforce discrimination, historically affecting education and employment opportunities. Fear of disclosure may discourage individuals from attending community-based events or accessing local campaigns. In such contexts, the anonymity of digital resources and the privacy of clinical settings often become more appealing.

In a stigmatized environment, patients prefer the relative anonymity of digital platforms and the confidentiality of a doctor’s office, avoiding community gatherings. Public health strategies should therefore focus on disseminating evidence-based messages within the digital platforms where women are already active, such as certified WeChat mini-programs [32], and on reducing stigma that discourages engagement with community resources. Addressing misconceptions and stigma is essential for improving the accuracy of public knowledge and reducing discrimination.

### Transmission misconceptions: a challenge for public health messaging and a driver of stigma

Misconceptions about transmission routes were common, with 41.9% of participants incorrectly believing that HBV can be transmitted through food. This misunderstanding may stem from confusion between HBV and HAV, as HAV is often discussed in the context of foodborne transmission. If health education does not clearly distinguish among types of viral hepatitis, these misconceptions may persist. Such misunderstandings have significant consequences. Erroneous beliefs about casual transmission can promote stigma and discrimination, discouraging individuals from seeking testing or disclosing their status [33, 34]. Addressing these myths is therefore essential not only for improving knowledge but also for reducing stigma and strengthening prevention [35]. Public messaging should explicitly clarify that HBV is not transmitted through food or water, highlight the actual transmission routes, and address the social implications of these misconceptions.

### Persistent knowledge gaps in a global and national context

Our finding that 41.9% of participants believed HBV could be transmitted by food and that only 60.1% correctly identified MTCT reflects a broader, globally documented challenge. Studies from Ghana and Pakistan have similarly reported substantial knowledge gaps among pregnant women, including limited awareness of MTCT [36, 37]. In China, disparities in HBV knowledge remain prevalent, particularly between urban and rural populations, where rural areas often exhibit poorer awareness [38, 39]. These patterns indicate that inadequate HBV literacy is a systemic barrier to elimination efforts.

As shown in previous research, limited knowledge is associated with reduced engagement in the HBV prevention cascade, including lower screening uptake, hesitancy toward antiviral therapy, and delays in neonatal immunization [23, 40]. Our study reinforces that HBsAg-positive pregnant women, who are central to MTCT interruption, remain vulnerable due to these persistent gaps.

### Determinants of knowledge: the overriding influence of occupation and residence

Our findings challenge the conventional view that formal education is the strongest determinant of health literacy [41]. Instead, occupation and place of residence were more closely associated with HBV knowledge. This should be interpreted carefully, however, in light of our study design. Because participants needed to complete the questionnaire independently, women with the lowest literacy levels were likely underrepresented. These women are also among the most vulnerable.

This selection effect may explain the absence of an association between education level and knowledge in our sample. Among women with at least basic literacy, environmental factors encountered in adulthood, including occupational settings and geographic access to healthcare, may play a more pronounced role in shaping HBV knowledge. These findings underscore the need for health education strategies tailored to specific occupational and geographic contexts rather than solely relying on formal education as a predictor [42]. Future studies should intentionally include women with lower literacy levels to better understand the full spectrum of HBV knowledge determinants and to design truly equitable educational interventions.

### Public health implications and action framework

Our results suggest that achieving the WHO and national goals for eliminating HBV MTCT by 2030 requires more than biomedical measures. Vaccination and antiviral therapy are critical but insufficient in isolation. Addressing disparities in health literacy and related social determinants, including work environment and urban-rural differences, is essential. The persistence of misconceptions, limited awareness of HBeAg status, and inadequate prior screening highlight gaps in current health education programs.

To address these systemic challenges, we propose a multi-level action framework that encompasses community education, healthcare provider training, digital health interventions, policy development, and improved access to screening and treatment.

#### Clinical level

Integrate comprehensive HBV serological testing, including HBsAg, HBeAg, and HBV DNA, and provide structured counseling in routine antenatal and preconception care.

#### Digital health level

Develop evidence-based digital education tools, such as official WeChat accounts and mini-programs, to counter misinformation and align with participants’ information-seeking behaviors.

#### Public messaging level

Implement campaigns that frame HBV screening as an essential component of women’s health and parental responsibility while addressing misconceptions, such as foodborne transmission, to reduce stigma.

#### Community and occupational level

Deliver targeted interventions in rural areas through trusted local providers and incorporate HBV education into workplace wellness programs in urban settings, particularly in industries with many female workers. Offering such programs during working hours or through flexible digital modules may increase participation.

These strategies aim to strengthen maternal knowledge, reduce stigma, and increase adherence to MTCT prevention across clinical, patient, and community levels.

## Limitations

This study has several limitations. First, the representativeness of the sample may be affected by selection bias. Although random sampling was used, the response rate was 49.3%, raising the possibility of non-response bias. Women who did not participate may have had lower HBV knowledge or health awareness, which could lead to an overestimation of knowledge levels in the study population. Additionally, recruitment occurred exclusively in tertiary hospitals within a single city. Women accessing these facilities may differ from those using primary care services in terms of socioeconomic status, health-seeking behavior, and clinical characteristics, which may limit generalizability.

Requiring participants to complete the questionnaire independently may have also introduced literacy and digital access bias. Women with the lowest literacy skills or limited digital competencies, who may also have the poorest HBV knowledge, might have been inadvertently excluded. The null finding between education and knowledge should therefore be interpreted cautiously. Among women with basic literacy, other factors such as occupation and residence may exert greater influence, but this does not diminish the importance of education in broader HBV health literacy.

Second, the cross-sectional design precludes causal inference regarding the associations identified.

Third, the relatively small sample size and the single-city setting may limit the generalizability of the findings to other parts of China, where HBV prevalence, health literacy, and healthcare infrastructure may vary.

Fourth, although the study identified a high rate of first-time diagnosis during pregnancy, the design and scope of the questionnaire limited our ability to explore determinants in depth, including detailed insurance status and other healthcare access metrics.

Fifth, self-reported data are subject to recall and social desirability biases, particularly for prior screening and information sources.

Sixth, broad occupational categories may have obscured differences in knowledge within heterogeneous groups, such as homemakers and unemployed individuals.

Seventh, the knowledge assessment focused on transmission and prevention but did not evaluate understanding of the full MTCT prophylaxis protocol, including antiviral therapy. This limitation may result in an overestimation of preparedness among at-risk pregnant women.

Finally, although designed for a broader parent study, the current analysis used only the knowledge, attitude, and practice components and did not incorporate biomarker data such as HBeAg or HBV DNA.

## Conclusion

China’s universal antenatal HBV screening policy has been effectively implemented and has identified large numbers of infected pregnant women, representing a critical step in eliminating MTCT. Our study demonstrates that the policy promotes equity by identifying disadvantaged groups, including those without health insurance and women living in rural areas, who are more likely to be diagnosed through this safety net.

Beyond identification, our findings indicate that maternal HBV knowledge in adulthood is shaped predominantly by concurrent environmental and socioeconomic factors. Occupation and place of residence were stronger predictors of HBV knowledge than formal education. Although universal screening successfully identifies HBsAg-positive pregnant women, significant knowledge gaps persist. These findings highlight the need for a strategic shift away from uniform health education approaches. Instead, future public health initiatives must be tailored to specific occupational groups and geographic contexts. Integrating such targeted interventions into the national prevention framework is essential for addressing disparities, engaging underserved populations, and achieving the 2030 MTCT elimination goals.

## Supplementary Information

Supplementary Material 1.

## Data Availability

All data generated and analyzed during this study are included in this published article. The raw data supporting the conclusions of this article will be made available by the authors, without undue reservation, to any qualified researcher. the main corresponding author Gaiyan Du should be contacted if someone wants to request the data in reasonable.

## Abbreviations

HBV: Hepatitis B Virus
MTCT: Mother-to-Child Transmission
HBsAg: Hepatitis B Surface Antigen
HBeAg: Hepatitis B e Antigen
WHO: World Health Organization
GDM: Gestational Diabetes Mellitus
KAP: Knowledge-Attitude-Practice
HIV: Human Immunodeficiency Virus
HCV: Hepatitis C Virus
HBIG: Hepatitis B Immunoglobulin
DNA: Deoxyribonucleic Acid
CI: Confidence Interval
OR: Odds Ratio
SD: Standard Deviation
NGO: Non-Governmental Organization
