# Supplementary material for: Knowledge, perceptions, and health education needs regarding hepatitis B virus among HBsAg-positive pregnant women in Taiyuan, China: a cross-sectional study
Source: BMC Public Health. 2025 Dec 20;26:315. doi: 10.1186/s12889-025-26019-3 (PMC12838502; doi:10.1186/s12889-025-26019-3)
Supplement: Supplementary file 1 — Supplementary Material 1. [file 12889_2025_26019_MOESM1_ESM.pdf]

2021 Taiyuan City Hepatitis B Mother-to-Child Transmission Blockade Effectiveness Evaluation Questionnaire

Dear Parents,

This survey requires you to fill out a questionnaire. We will also collect a venous blood sample from the newborn for free quantitative testing of hepatitis B surface antibodies.
We guarantee strict confidentiality for all questions in this survey that may involve your personal and your child's privacy. After the survey is completed. Data will be securely deleted to prevent any leakage..
This project follows the principle of complete voluntariness.
If you agree, please sign below. Thank you!

Your Name: (The parturient woman's own name) _________________________

1.Date of Form Completion: _________________________

2.Your Mobile Phone Number: _________________________

3.Your Education Level: (Please check one ☐)
○ Primary school and below
○ Junior high school
○ High school / Technical secondary school / Vocational high school
○ Technical college / Associate degree
○ Bachelor's degree
○ Master's degree and above

4.Your Age: _________

5.Your Occupation: (Please check one ☐)
○ Teacher
○ Commercial services
○ Farmer
○ Worker
○ Government official/Cadre
○ Medical staff
○ Homemaker and unemployed
○ Other _________

6.Monthly Income Per Capita: (Please check one ☐)
○ _________ Yuan/Month
○ Unknown

7.Place of Residence: (Please check one ☐)
○ Urban
○ Urban village
○ Rural

8.Health Insurance Type: (Please check one ☐)
○ Health insurance
○ Self-pay

9.Time when you first discovered you had hepatitis B: ( ) Year (e.g., 2000) __________________________

10.If you discovered you had hepatitis B during this pregnancy, when exactly was it: (Please check one ☐)
○ Early pregnancy check-up
○ Late pregnancy check-up
○ Surgical examination

11.Which items from your hepatitis B serology panel (five markers) were abnormal? (Please check all that apply ☐)
□ Surface antigen (HBsAg)
□ Surface antibody (Anti-HBs)
□ e antigen (HBeAg)
□ e antibody (Anti-HBe)
□ Core antibody (Anti-HBc)

12.Are you? (Please check one ☐)
○ "Large Three Positive" (HBsAg+, HBeAg+, Anti-HBc+)
○ "Small Three Positive" (HBsAg+, Anti-HBe+, Anti-HBc+)
○ Not sure

13.Your liver function indicators: (Please check one ☐)
○ Normal
○ Abnormal
○ Not sure

14.Do any of your parents or siblings have hepatitis B? (Please check one ☐)
○ Yes
○ No
○ Not sure

The following relates to information about this delivery: (If you have not yet given birth, you may complete this section after delivery)

15.This is your ( )th delivery __________________________

16.Hospital where delivery took place: __________________________

17.Gestational week at delivery: ( ) weeks __________________________

18.Date of Birth: ____ Year ____ Month ____ Day ____ Time of birth (24-hour format) __________________________

19.Method of delivery for this birth: (Please check one ☐)
○ Vaginal delivery
○ Cesarean section

20.If delivered vaginally, was there perineal tearing? (Please check one ☐)
○ Yes
○ No

The following questions ask about your knowledge of hepatitis B:

21.Through which channels do you typically obtain knowledge about hepatitis B? (Please check all that apply ☐)
□ Asking hospitals/doctors
□ Searching via Baidu/the internet
□ Searching via social media
□ Asking relatives and friends
□ Searching via magazines or books
□ Participating in NGO activities
□ Attending lectures and consultations organized by the community

22.In your opinion, which of the following are transmission routes for hepatitis B? (Please check all that apply ☐)
□ Receiving blood transfusions and blood products
□ Sharing syringes/needles with intravenous drug users
□ Sharing towels/toothbrushes/utensils/razors
□ Unprotected sexual intercourse
□ Mother transmitting to her infant

23.In your opinion, which of the following are NOT transmission routes for hepatitis B? (Please check all that apply ☐)
□ Shaking hands with a hepatitis B infected person/patient
□ Kissing
□ Eating at the same table
□ Mosquito bites

24.In your opinion, which of the following measures can protect others from hepatitis B infection? (Please check all that apply ☐)
□ Actively getting vaccinated against hepatitis B
□ Not sharing razors, toothbrushes with others
□ Washing hands before meals and after using the toilet, maintaining good hygiene habits

25.Before pregnancy, did you proactively get tested for liver function and/or hepatitis B markers? (Please check one ☐)
○ Yes
○ No

26.Have your family members been vaccinated against hepatitis B? (Please check one ☐)
○ Yes
○ No

27.Before pregnancy, did you have a habit of regular physical exercise? (Please check one ☐)

○ Yes
○ No

28.Before pregnancy, did you have a smoking habit? (Please check one ☐)
○ Yes
○ No

29.Before pregnancy, did you have a drinking habit? (Please check one ☐)
○ Yes
○ No

30.In your opinion, is hepatitis B highly contagious? (Please check one ☐)
○ Highly contagious
○ Relatively contagious
○ Moderately contagious
○ Slightly contagious
○ Not contagious

31.In your opinion, how severe is the health harm caused by hepatitis B? (Please check one ☐)
○ Very severe
○ Relatively severe
○ Moderate
○ Not severe
○ Harmless

32.Which of the following are transmission routes for hepatitis B?(Please check all that apply ☐)

○Blood-borne transmission
○ Mother-to-child transmission
○ Sexual transmission
○Airborne transmission
○ Food-borne transmission

○ Some other transmission routes
